# Immune-Enhancing Effects of *Limosilactobacillus fermentum* in BALB/c Mice Immunosuppressed by Cyclophosphamide

**DOI:** 10.3390/nu15041038

**Published:** 2023-02-19

**Authors:** SukJin Kim, Hwan Hee Lee, Chang-Ho Kang, Hyojeung Kang, Hyosun Cho

**Affiliations:** 1 Department of Bio-Health Convergence Major, Duksung Women’s University, Seoul 01369, Republic of Korea; 2 Department of Pharmacy, Duksung Women’s University, Seoul 01369, Republic of Korea; 3 Duksung Innovative Drug Center, Duksung Women’s University, Seoul 01369, Republic of Korea; 4 Mediogen, Co., Ltd., Bio Valley 1-Ro, Jecheon-si 27159, Republic of Korea; 5 College of Pharmacy, Vessel-Organ Interaction Research Center, VOICE (MRC), Cancer Research Institute, Kyungpook National University, Daegu 41566, Republic of Korea

**Keywords:** immune-enhancing effects, *L. fermentum*, cyclophosphamide

## Abstract

This study evaluates the immune-enhancing effects of *Limosilactobacillus fermentum* on cyclophosphamide (CP)-induced immunosuppression in BALB/c mice. In vitro, the expressions of pro-inflammatory cytokines and MAPK signaling molecules in Raw264.7 cells were analyzed by ELISA and Western blot analysis. Moreover, cell proliferation, surface receptor expression, and cytotoxicity of NK-92 cells were examined by Cell Counting Kit-8, CytoTox96 assay, and flow cytometry, respectively. To investigate the immune-enhancing effects of selected *L. fermentum* strains in vivo, these strains were orally administered to BALB/c mice for 2 weeks, and CP was intraperitoneally injected. Then, liver, spleen, and whole blood were isolated from each animal. Administration of single *L. fermentum* strains or their mixture sustained the spleen weight, the counts of white blood cells compared to non-fed group. Splenocyte proliferation and NK cytotoxicity were significantly increased in all *L. fermentum*-fed groups. The frequency of B220+ cells was also significantly enhanced in splenocytes isolated from *L. fermentum* groups. In addition, the production of cytokines (TNF-α, IFN-γ) and antibodies was recovered in splenocyte supernatants isolated from *L. fermentum* groups. In conclusion, *L. fermentum* could be a suitable functional food additive for immune-enhancing effect.

## 1. Introduction

Cyclophosphamide (CP) is an alkylated anti-tumor drug that disrupts DNA replication and inhibits cell proliferation [1]. Therefore, CP is widely used as a chemotherapeutic agent for various cancers such as lymphoma, leukemia, breast cancer, and small-cell lung cancer and as a treatment for autoimmune diseases [2]. However, CP often results in serious side effects such as leukopenia, myelosuppression, immunosuppression, and cytotoxicity [3]. High doses of CP reduce body weight, relative weight of spleen and thymus, leukocyte and natural killer (NK) cell activity, and absolute numbers of B and T cells [4]. Depending on immunosuppression, patients may be at risk for various infections and complications, which delay diagnostic and treatment outcomes. To reduce the side effects of anticancer drugs, it is necessary to find safe immunomodulators, and functional foods with immunostimulatory properties.

Probiotics are generally defined as live microbial additives that provide health benefits to the host animal by improving the balance between the microbes in the gut [5]. Strains of *Bifidobacterium* and *Limosilactobacillus* are the most common probiotics and promote human health through characteristics such as safety privilege, antiviral activity, and genome-encoded host-interacting factors [6]. Reported beneficial effects of probiotic strains include reducing the symptoms of or preventing allergic diseases, affecting cholesterol levels, regulating the composition of the gut microbiome, and modulating the immune response of the host [7,8]. In mice, these strains significantly improve the mucosal immune responses through an increase in expression of TLR2 and IFNγ mRNA in the intestine and increase the synthesis of polyclonal IgG, IgM, and IgA in blood [9,10]. Administration of lactobacilli increases cytokine and immunoglobulin production, and activates immune-related cells (phagocytes and NK cells) [11]. The use of probiotics to improve the immune response of immunocompromised patients has received steady attention in recent years. Probiotics can improve immunity without side effects.

The immune system is an organization of cells and molecules that defends the body from infections and is composed of innate immunity and adaptive immunity [12]. Bone marrow, spleen, thymus, and immune cells including macrophages, spleen cells, neutrophils, and NK cells are essential for the immune function of the human body [13]. Innate immunity acts as the primary defender against external pathogens with an immediate response mediated by the activities of macrophages, dendritic cells, monocytes, neutrophils, and NK cells [14]. The adaptive immune system is regulated through the activation of B and T cells against antigens and is regulated by immunoglobulins, chemokines, and cytokines, including tumor necrosis factor-α (TNF-α), interferon (IFN-γ), and interleukin (IL)-6 [15]. Probiotics stimulate the host’s immune system and could be a potential treatment alternative to chemotherapy.

In the present study, we aimed to investigate the immune-enhancing effects of *Limosilactobacillus fermentum* strains in vitro and in vivo. We screened the effects of seven *Bifidobacterium* or *Limosilactobacillus* strains on the production of pro-inflammatory cytokines and the expression of mitogen-activated protein kinase (MAPK) signaling molecules in RAW 264.7 cells. We also examined the effects of *Bifidobacterium* or *Limosilactobacillus* strains on cell proliferation, surface receptor expression, and cytotoxicity of NK-92 cells. In addition, we investigated the immune-enhancing effects of selected *L. fermentum* strains on CP-induced immunosuppression in BALB/c mice and examined the immune-enhancing effects of *L. fermentum* in terms of weight change of body and organ, splenocyte proliferation, the frequency of lymphocyes, the production of cytokines and antibodies.

## 2. Materials and Methods

### 2.1. Sample Preparation

The following seven bacterial strains were supplied by Mediogen Co., Ltd. (Jecheon, Republic of Korea): *B. bifidum* MG731, *B. lactis* MG741, *L. fermentum* MG4538, *L. fermentum* MG5091, *L. fermentum* MG5159, *L. paracasei* MG5015, *L. reuteri* MG505 [Table 1].

For in vitro study, each strain was activated by culturing in MRS medium at 37 °C for 18 h. The bacterial suspension of the selected strain was diluted to an initial absorbance at 600 nm (OD600) of 1.0. Cell-free supernatants were obtained from bacterial cultures by centrifugation at 5000× *g* for 5 min and filtered through a 0.22 μm filter. The CFS (cell free supernatants) were lyophilized and stored at −20 °C. The lyophilized powder was dissolved in a medium, filtered through a 0.2 μm filter, and 5 mg/mL was used for experiments.

For in vivo study, each strain was activated by culturing in MRS broth. To prepare *Bifidobacterium* and *Limosilactobacillus* powder, freshly harvested bacterial pellets were mixed well with the cryoprotectant mixture, lyophilized, and stored at 4 °C until use.

### 2.2. Cell Culture

RAW 264.7 cells were cultured in Dulbecco’s modified Eagle’s medium (DMEM, Gibco, Grand Island, NY, USA) supplemented with 10% heat-inactivated fetal bovine serum (FBS; Young-In Frontier, Seoul, Korea). NK-92 cells were cultured in alpha minimum essential medium (αMEM, Gibco) supplemented with 20% FBS, 0.1 mM 2-mercaptoethanol, and rIL-2. Primary splenocytes isolated from BALB/c mice were cultured in Roswell Park Memorial Institute (RPMI) 1640 medium with L-glutamine (Corning Inc., NY, USA). All cells were cultured in the presence of 1% penicillin and streptomycin (Gibco) at 37 °C in a humidified atmosphere with 5% CO_2_.

### 2.3. ELISA

RAW 264.7 cells were seeded at a density of 1 × 10^6^ cells/well in a 6-well flat-bottom plate and concentrations of bacterial strains (5 mg/mL) were added for 24 h. Cell-free supernatants were harvested to measure the production of TNF-α, IL-6, and IL-1β using a mouse ELISA kit (BD Biosciences, East Rutherford, NJ, USA). Absorbance was measured at 450 nm using a microplate reader (BMG Lactobacillustech, Ortenberg, Germany).

### 2.4. Western Blot Analysis

RAW 264.7 cells were seeded and treated with bacterial strains as in *2.3*. Cells were lysed with protein extraction buffer (Intron Biotechnology, Seoul, Korea). Proteins in cell lysates were quantified by the Bradford assay, separated by polyacrylamide gel electrophoresis, and transferred to nitrocellulose membranes, which were then incubated with primary antibodies against Erk (#9102), pErk (#4307), GAPDH (#5174) and then with secondary anti-rabbit IgG (#7074) and anti-mouse IgG (#7076), all obtained from Cell Signaling Technology (CST, Danvers, MA, USA) [16]. Anti-NF-κB (ab194726), anti-pNF-κB (ab16502), and anti-TLR2 (ab108998) antibodies were obtained from Abcam (Cambridge, UK). Proteins were visualized by enhanced chemiluminescence detection (ECL System, Bio-Rad, Hercules, CA, USA) and quantified using the ImageJ program (National Institutes of Health, Bethesda, MD, USA). Expression levels relative to that of GAPDH were determined.

### 2.5. NK-92 Cell Proliferation

The proliferative effects of bacterial strains on NK-92 cells were assessed by using a Cell Counting Kit-8 (CCK-8, Dojindo Laboratories, Kumamoto, Japan). NK-92 cells were seeded at a density of 5 × 10^4^ cells/well in a 96-well flat-bottom plate and different concentrations of bacterial strains (5 mg/mL) were added for 24 h [17]. Then, CCK-8 solution was added to each well, and the cells were incubated in accordance with the reactive time of the solution. Absorbance was measured at 450 nm using a microplate reader.

### 2.6. In vitro NK Cytotoxicity Assay

The cytotoxic effect of NK-92 cells on K562 cells was assessed using a CytoTox96 Non-radioactive Cytotoxicity Assay Kit (Promega, Wisconsin, USA). Briefly, NK-92 cells (effector cells, E) were plated at a density of 1 × 10^5^ cells/well in a 96-well plate with bacterial strains (5 mg/mL) and incubated for 24 h [17]. The target K562 cells (T) were cocultured with E, cells at an E/T ratio of 1:2 for 4 h. The cells were centrifuged at 1600*× g* for 4 min, and the supernatants were transferred into a fresh plate. Then, CytoTox96 reagent was added to each well and incubated for 30 min at room temperature in the dark, and finally stop solution was added to each well. Within 1 h, absorbance was measured at 490 nm using a microplate reader.

### 2.7. Fluorescent Antibody and Cell Surface Antigen Staining

NK-92 cells were stained with antibodies against CD56 and NKG2D (both from BD Biosciences) according to the manufacturer’s instructions. After staining, cells were analyzed by flow cytometry (Novocyte Flow Cytometer, ACEA Biosciences, USA). The positivity for CD56 and NKG2D was determined by comparison with the defined cutoff values obtained with unstained control cells, as previously described.

### 2.8. Analysis of Short-Chain Fatty Acids (SCFAs) Present in the Culture Medium

The SCFAs in the fermented broth were analyzed via gas chromatography–mass spectrometry (QP2020 NXW/ORP230; Shimadzu, Kyoto, Japan) using the headspace solid-phase microextraction method (Thitiratsakul and Anprung 2014) with minor modifications. The samples were separated using a Stabilwax-DA column (60 m × 0.32 mm × 0.25 μm, Shimadzu). Analytical conditions were as follows: oven temperature was held at 50 °C for 2 min, and raised to 100 °C at 10 °C/min, 200 °C at 2 °C/min, increased to 220 °C at 20 °C/min, and maintained for 2 min; the splitless mode was used; helium was used as the carrier gas at the flow of 2 mL/min. The mass spectrometer was operated in the electron-impact mode at 65 eV. The scan range was 40–200 m/z, the scan rate was 0.2 s/scan, and the electron energy was 70 eV. The ionization source and quad temperature were at 200 °C and 150°C, respectively. Each extracted sample peak area was normalized to the initial volume of the sample following quantification. Linear regression equations for each analyte were calculated with the calibration curve of the peak area versus analyte concentrations (µmol).

### 2.9. Immunosuppression in BALB/c Mice by Cyclophosphamide

All the animal experiments were conducted in accordance with the recommendations in the National Research Council’s Guide for the Care and Use of Laboratory Animals. The experimental protocol was approved by the Animal Experiments Committee of Duksung Women’s University (permit number: 2022-003-007). BALB/c mice (male, 5 weeks old) were obtained from RaonBio Co. Ltd. (Seoul, Korea). Mice were adapted for 7 days before the experiment and maintained under a controlled temperature (23 ± 2 °C) with a 12 h/12 h light/dark cycle. Mice were randomly divided into seven groups (*n* = 6): (1) Control group: drinking water; (2) CP only group: drinking water, (3) RG group: red ginseng (10 mg/kg) for positive control; (4) MG4538 group: *L. fermentum* MG4538 (1 × 10^9^ CFU/mouse); (5) MG5091 group: *L. fermentum* MG5091 (1 × 10^9^ CFU/mouse); (6) MG5159 group: *L. fermentum* MG5159 (1 × 10^9^ CFU/mouse); (7) 3Mix group: 1:1:1 mixture of *L. fermentum* MG4538, MG5091, and MG5159 (1 × 10^9^ CFU/mouse in total). After 2 weeks of feeding, mice of all groups except the control were intraperitoneally injected with CP at a dose of 150 and 100 mg/kg/day on days 14 and 16, respectively [18]. Body weight changes were checked daily until the end of the experiment. Mice were sacrificed 5 days after the second CP dose, and the liver and spleen were removed and weighed.

### 2.10. Complete Blood Cell Count

Five days after the second injection of CP, blood was collected to analyze complete blood cell count (CBC). White blood cell (WBC), differential white cell, and red blood cell (RBC) counts, hemoglobin concentration (HGB), platelet count (PLT), mean corpuscular volume (MCV), mean corpuscular hemoglobin concentration (MCHC), and mean corpuscular hemoglobin (MCH) were measured with an automated hematology analyzer (XN-100, Sysmex, Kobe, Japan).

### 2.11. In Vivo NK Cytotoxicity Assay

Primary splenocytes were harvested from each animal and the cytotoxic effect of NK cells on YAC-1 cells was measured using a CytoTox96 Non-radioactive Cytotoxicity Assay Kit. Briefly, YAC-1 cells (target cells) were plated at a density of 5 × 10^5^ cells/well in a 96-well plate and were cocultured with splenocytes (effector cells), at an E/T ratio of 1:5 for 4 h [19]. The cells were centrifuged at 1600*× g* for 4 min, and the supernatants were transferred into a fresh plate. Then, CytoTox96 reagent was added to each well and incubated for 30 min at room temperature in the dark, and finally stop solution was added to each well. Within 1 h, absorbance was measured at 490 nm using a microplate reader.

### 2.12. Fluorescent Antibody and Cell Surface Antigen Staining

The subpopulations (CD4+ or CD8+) of T cells and B cells from the spleen were measured. Cell Surface Antigen Staining Splenocytes were stained with anti-mouse CD4-PE, anti-mouse CD8-APC and anti-mouse B220-PE (BD Biosciences) according to the manufacturer’s instructions. After staining, cells were analyzed by flow cytometry (Novocyte Flow Cytometer). The positivity for CD4, CD8 and B220 was determined by comparison with the defined cutoff values obtained with unstained control cells as previously described.

### 2.13. Splenocyte Proliferation Assay

Proliferative effect of bacterial strains on primary mouse splenocytes was assessed using a CCK-8 kit. Cells were seeded at a density of 1 × 10^5^ cells/well in a 96-well flat-bottom plate and incubated for 72 h. Then, CCK-8 solution was added to each well, and the cells were incubated in accordance with the reactive time of the solution. Absorbance was measured at 450 nm using a microplate reader.

### 2.14. Splenocyte Cytokine Production

Effect of bacterial strains on cytokine production by primary mouse splenocytes was assessed. Cells were seeded at a density of 1 × 10^6^ cells/well in a 6-well flat-bottom plate with or without 10 μg/mL of Concanavalin A (ConA) and incubated for 72 h. Then, cell-free supernatants were harvested to measure the production of TNF-α and IFN-γ using a mouse ELISA kit (BD Biosciences). Absorbance was measured at 450 nm using a microplate reader.

### 2.15. H&E Staining and Immunohistochemistry (IHC)

Intestinal tissue was harvested from mice to perform IHC. Intestinal tissue paraffin blocks were fabricated and cut at a thickness of 4 μM. Sections were deparaffinized and rehydrated using xylene, 100% ethanol, 90% ethanol, and 80% ethanol. Intestinal tissue were stained with hematoxylin and eosin stain (H&E). For IHC, tissues were blotted with primary antibodies in PBST overnight at 4 °C. Occludin (#91131) and ZO-1 (#sc-33725) were purchased from Cell Signaling Technology (CST; Danvers, MA, USA) and Santa Cruz Biotechnology (Dallas, TX, USA) respectively. After treatment, cells were treated with a secondary antibody, mouse anti-rabbit IgG-HRP (#sc-2537) for 2 h at room temperature. Then, the slices were stained with DAB (Vector Lactobacillus oratories, Burlingame, CA, USA) and observed under a microscope (400×).

### 2.16. Statistical Analysis

All in vitro data were analyzed in triplicate and are presented as mean ± standard deviation (SD) using *t*-test. In vivo data were analyzed from six animals per group. Data were statistically analyzed using one-way analysis of variance (ANOVA) with Duncan’s multiple range test. Data were analyzed using SPSS version 22 (IBM Corp., Armonk, NY, USA). *p* < 0.05 was regarded as statistically significant.

## 3. Results

### 3.1. Effects of Bifidobacterium and Limosilactobacillus on Pro-Inflammatory Cytokine Production and the Expression of MAPK Signaling Molecules in RAW 264.7 Cells

**Figure 1:**
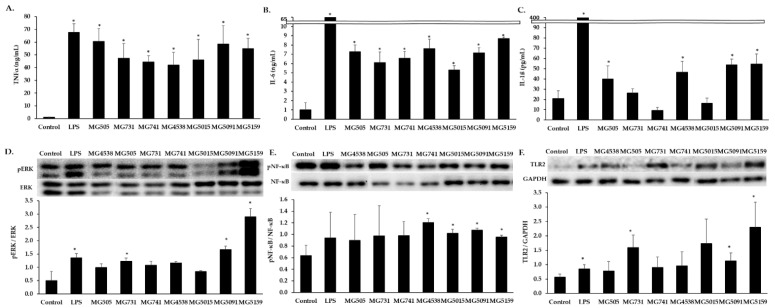
Effects of *Bifidobacterium* and *Limosilactobacillus* on pro-inflammatory cytokine production and the expression of MAPK signaling molecules in RAW 264.7 cells. Cells were incubated with *Bifidobacterium* or *Limosilactobacillus* (5 mg/mL) or lipopolysaccharide (LPS; 1 µg/mL) for 24 h and culture supernatants were harvested. Production of (**A**) TNF-α, (**B**) IL-6, and (**C**) IL-1β was measured by enzyme-linked immunosorbent assay. To examine MAPK signaling pathways, the following ratios were determined by Western blot analysis: (**D**) pErk/Erk, (**E**) pNF-κB/NF-κB and (**F**) TLR2/GAPDH. All data are means ± SD from three independent experiments. * *p* < 0.05 vs. control cells.

First, we used RAW 264.7 cells to investigate the effects of Bifidobacterium and Lactobacillus on innate immunity. The production of pro-inflammatory cytokines was measured after stimulation with bacterial strains (5 mg/mL) or lipopolysaccharide LPS (1 µg/mL) vs. untreated control (Figure 1A–C). There was a dramatic increase in TNF-α and IL-6 production after treatment with all bacterial strains examined. Secretion of IL-1β was significantly increased by MG4538, MG5091, MG5159, and MG505 vs. untreated control.

The expression of nuclear factor kappa-light-chain-enhancer of activated B cells (NF-κB), pNF-κB, extracellular-signal-regulated kinase (Erk), pErk and TLR-2 were examined in RAW 264.7 cells treated with or without *Bifidobacterium* or *Limosilactobacillus*. The level of pErk was increased by all bacterial strains, significantly so by MG731, MG5091, and MG5159 (Figure 1D). The level of pNF-κB was significantly increased by MG4538, MG5015, MG5091, and MG5159 and tended to be increased by the other three strains (Figure 1E). The expression of TLR2 was significantly increased by MG731, MG5091, and MG5159 (Figure 1F).

### 3.2. Effect of Bifidobacterium and Limosilactobacillus on NK-92 Cells

**Figure 2:**
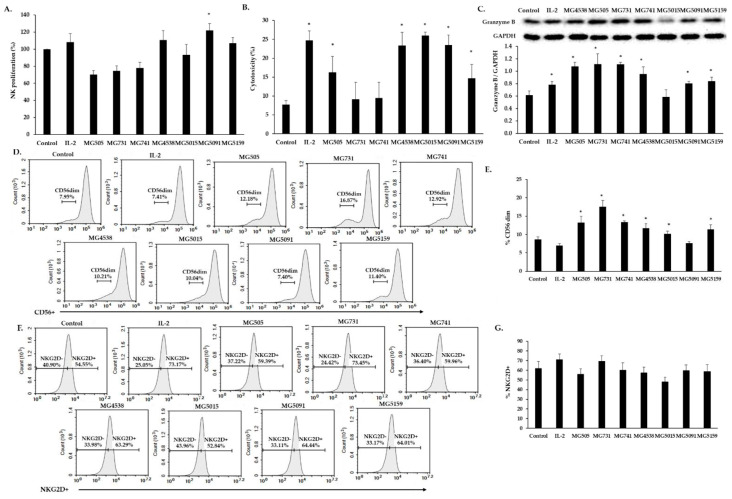
Effect of *Bifidobacterium* and *Limosilactobacillus* on NK-92 cells. (**A**) NK-92 cell proliferation determined using CCK-8 assay. (**B**) Cytotoxicity of NK-92 cells against K562 cells. (**C**) Secretion of granzyme B. (**D**,**F**) Representative FACS plots showing CD56+ and NKG2D+ expression. (**E**) and (**G**) Percentage of CD56+ and NKG2D+ expression. All data are means ± SD from three independent experiments. * *p* < 0.05 vs. control cells.

Treatment with MG5438, MG5091, and MG5159 increased proliferation of NK-92 cells in comparison with the untreated cells (Figure 2A). In cytotoxicity assay (Figure 2B), NK cytotoxicity against K562 cells was significantly increased by treatment with MG505, MG4538, MG5015, MG5091, and MG5159. The secretion of granzyme B (GrzB) from NK-92 cells, as analyzed by Western blotting, was significantly increased by treatment with all bacterial strains except MG5015 (Figure 2C).

Treatment of NK-92 cells with all strains except MG5091 significantly increased the frequency of the CD56dim cells in comparison with NK-92 single culture (Figure 2D,E). However, none of the strains tested significantly affected NKG2D expression in NK-92 cells (Figure 2F,G).

### 3.3. Analysis of SCFAs in L. fermentum Present in the Culture Medium

The SCFA profile of *L. fermentum* MG4538, MG5091, and MG5159 and their concentrations is shown in Table 2. The average acetic acid concentration of *L. fermentum* MG4538, MG5091, and MG5159 was 4286.3 mg/L. The propionic acid concentrations of *L. fermentum* MG4538, MG5091, and MG5159 were 2.07, 2.02, and 1.84 mg/L, respectively. The concentration of butyric acid in *L. fermentum* was more than 3.4 mg/L.

There was no significant difference between the short-chain fatty acid concentrations for each *L. fermentum*.

### 3.4. Effect of L. fermentum on the Weight Changes of Body, Liver and Spleen in CP-Treated Immunosuppressed Mice

**Figure 3:**
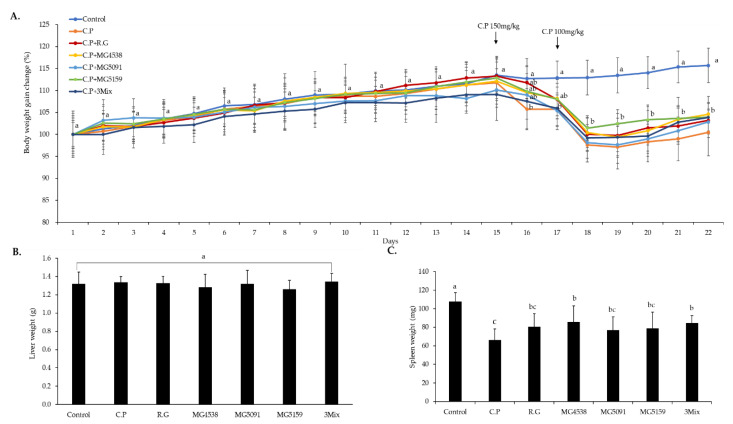
Effects of *L. fermentum* on body and organ weight changes in cyclophosphamide (CP)-treated immunosuppressed mice. Mice were intraperitoneally injected with CP (150 and 100 mg/kg) after administration of drinking water (control and CP-only groups), red ginseng (RG) extract (10 mg/kg), *L. fermentum* MG4538 (1 × 10^9^ cells/mouse), *L. fermentum* MG5091 (1 × 10^9^ cells/mouse), *L. fermentum* MG5159 (1 × 10^9^ cells/mouse), or a mixture of MG4538, MG5091, and MG5159 (3 × 10^9^ cells/mouse in total). (**A**) Whole body weight change was monitored daily until the experimental end point. (**B**) Liver and (**C**) spleen weight. All data are means ± SD (*n* = 6) and were analyzed with one-way ANOVA to compare differences between groups for each item. Different letters indicate significant difference between means at *p* < 0.05.

A steady increase in whole body weight in all groups was recorded until 14 days (Figure 3A), indicating no general toxicity by oral administration of *L. fermentum*. After CP injection, body weights of all CP-treated groups were significantly decreased in comparison with the control group. Liver weight did not differ among the groups (Figure 3B), but spleen weight was significantly higher in the groups administered with *L. fermentum* than in the CP-only group (Figure 3C).

### 3.5. Effect of L. fermentum on CBC in CP-Treated Mice

We observed a significant reduction in WBC, LWP, RBC, HGB, HCT, MCV, RDW, and platelet levels in the CP-only group in comparison with the control group (Table 3). Treatment with RG, MG5091, and 3Mix significantly increased WBC count in comparison with the CP-only group. A significant increase in the level of lymphocytes was observed in all treatment groups (≥40.52%) in comparison with the CP-only group (35.30%). Platelet count was significantly higher in all treatment groups (≥517.00 × 10^3^ cells/μL) in comparison with the CP-only group (224.83 × 10^3^ cells/μL). The *L. fermentum* treatment groups had significantly higher RBC, HGB, and HCT levels than the control group. Taken together, these data indicate that the administration of CP reduced WBC, platelet, and HGB levels and *L. fermentum* treatment rescued these levels.

### 3.6. Effects of L. fermentum on the Frequency of T Cells and Cytotoxicity of NK Cells in CP-Treated Immunosuppressed Mice

**Figure 4:**
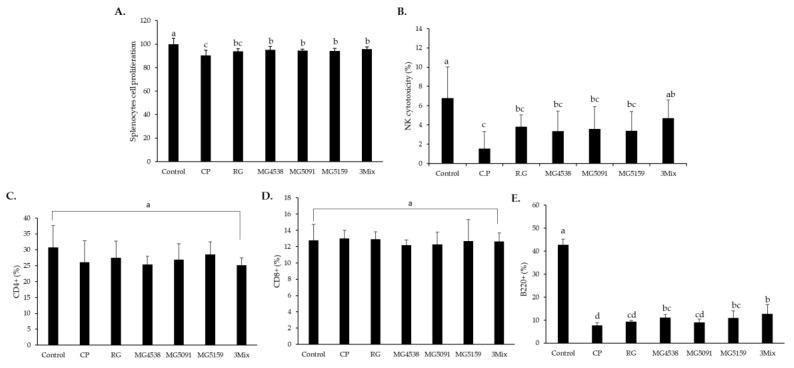
Effect of *L. fermentum* on the frequency of T cells and cytotoxicity of NK cells in CP-treated immunosuppressed mice. (**A**) Splenocyte proliferation, (**B**) cytotoxicity of NK cells against YAC-1 cells. Frequencies of (**C**) CD4+, (**D**) CD8+, and (**E**) B220+ cells were analyzed using flow cytometry. All data are means ± SD (*n* = 6) and were analyzed with one-way ANOVA to compare differences between groups for each item. Different letters indicate significant difference between means at *p* < 0.05.

The proliferation of splenocytes from the CP group (90.5%) was significantly suppressed in comparison with that from the control group. However, the RG group and all *L. fermentum* treatment groups showed a significant increase in splenocytes proliferation (RG, 93.9%; MG438, 95.1%; MG5091, 94.6%; MG5159, 94.4%; 3Mix, 95.9%) (Figure 4A). NK cytotoxicity against YAC-1 cells was significantly lower in the CP group than in the control group (Figure 4B). RG, MG4538, MG5091, MG5159, and 3Mix groups had higher cytotoxicity in comparison with the CP group, and the effects RG and 3Mix were particularly significant. The percentages of CD4+ and CD8+ T cells were not significantly different among the groups (Figure 4C,D), although that of CD4+ T cells decreased from 30.8% in the control group to 26.1% in the CP group (Figure 4C). The frequency of CD4+ T cells was 25.4%, 27.0%, and 28.5% in the order of MG4538, MG5091, and MG5159. The frequency of B220+ cells was significantly recovered in the RG group (9.3%) and *L. fermentum* treatment groups (MG4538, 11.1%; MG5091, 9%; MG5159, 10.9%; 3Mix, 12.6%) than CP group (7.7%) (Figure 4E).

### 3.7. Effect of L. fermentum on the Production of Cytokines (TNF-α, INF-γ) and Antibodies (IgM, Total IgG) in CP-Treated Immunosuppressed Mice

**Figure 5:**
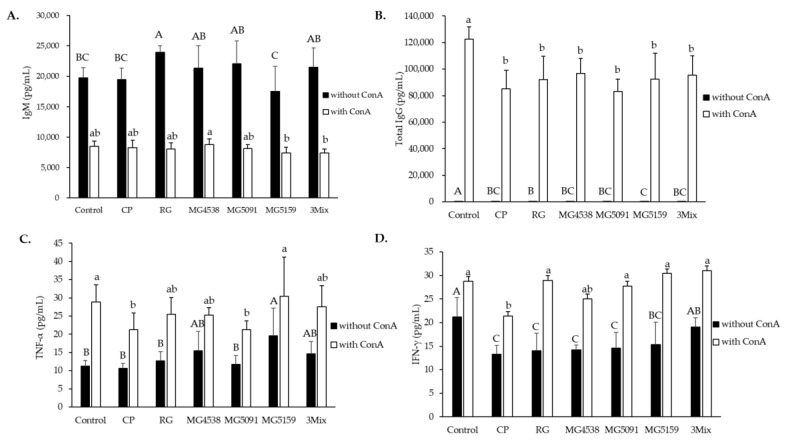
Effect of *L. fermentum* on splenocyte cytokines (TNF-α, IFN-γ) and antibodies (IgM, total IgG) in CP-treated immunosuppressed mice. Primary mouse splenocytes were seeded in a 6-well flat-bottom plate with or without Con A (10 μg/mL) and incubated for 72 h. Then ELISA kits were used to measure the production of (**A**) IgM, (**B**) IgG, (**C**) TNF-α, and (**D**) IFN-γ. All data are means ± SD (*n* = 6) and were analyzed with one-way ANOVA to compare differences between groups for each item. Different letters indicate significant difference between means at *p* < 0.05: uppercase, comparisons among cells with ConA; lowercase, comparisons among cells without ConA.

The effects of *L. fermentum* on the secretion of cytokines and antibodies in the immunosuppressed mice were examined using supernatants harvested from splenocyte cultures. ConA treatment decreased IgM expression and increased IgG expression (Figure 5A,B).

Regardless of ConA, CP injection significantly decreased the expression of TNF-α, IFN-γ and total IgG in comparison with that in the control group. TNF-α expression with ConA was significantly higher in the RG (25.55 ± 4.52 pg/mL), MG4538 (25.29 ± 2.01 pg/mL), MG5159 (30.41 ± 10.80 pg/mL), and 3Mix (27.53 ± 5.86 pg/mL) groups than in the CP group (21.31 ± 4.52 pg/mL). Administration of RG and *L. fermentum*, especially 3Mix, restored IFN-γ expression to the levels similar to that in the control group.

### 3.8. Effect of L. fermentum on the Intestinal Mucosal Barrier in CP-Treated Immunosuppressed Mice

**Figure 6:**
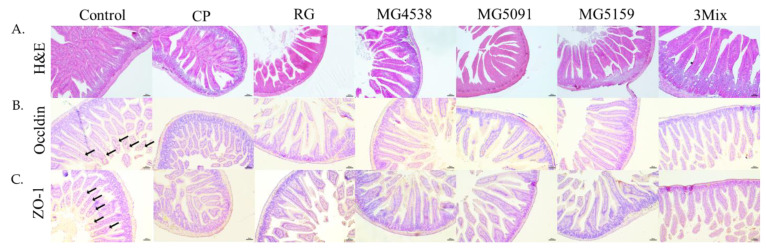
Effect of *L. fermentum* on the intestinal mucosal barrier in immunosuppressed mice. (**A**) H&E staining. Immunohistochemistry of (**B**) occludin and (**C**) ZO-1. Scale bars, 100 px. Black arrows indicate the dots of occludin and ZO-1 (magnification 400×).

H&E staining of ileum tissues showed that the intestines of mice in the control group had normal glands and slender villi with a complete structure and tight arrangement (Figure 6A). In contrast, the intestinal wall in the CP group was severely damaged, and the villi were short and even detached. Administration of *L. fermentum* did not recover small intestine tissue damage. Immunohistochemistry data revealed the expression of occludin and ZO-1 in the control group, its absence in the CP-only group, and no recovery in any of the other groups.

## 4. Discussion

In the present study, we demonstrated the immune-enhancing effects of *Bifidobacterium* and *Limosilactobacillus* strains in CP-induced immunosuppressed mice. Many studies have reported that probiotics regulate innate and adaptive immune responses by stimulating dendritic cells, macrophages, and T and B lymphocytes [20]. Treatment with probiotics also activates NK cells [21]. However, the immune-enhancing effects of *L. fermentum* and their mechanisms have not been investigated in CP-induced immunosuppressed mice. Our study is the first report that *L. fermentum* has significant immune-enhancing effects in these mice.

We first investigated the effects of *Bifidobacterium* and *Limosilactobacillus* on macrophages using RAW 264.7 cells. In previous studies, *Limosilactobacillus* strains were not cytotoxic at 5 mg/mL to RAW 264.7 cells [22], so we used the same concentration in in vitro experiments. When macrophages are exposed to stimuli, they secrete cytokines such as TNF-α, IL-6, IL-1β, and INF-γ [23]. The main function of cytokines is to regulate inflammation, and they play an important role in modulating the immune response [24]. TNF-α and IL-6 production was dramatically increased in RAW 264.7 cells treated with all bacterial strains or LPS (1 µg/mL) in comparison with untreated controls (Figure 1A,B). The secretion of IL-1β was increased the most in RAW 264.7 cells treated with *L. fermentum* MG4538, MG5091, and MG5159 (Figure 1C). The production of cytokines is regulated by MAPK pathway activation [25]. The expression level of TLR2 and the phosphorylation levels of Erk and NF-κB were increased by all the seven bacterial strains tested in comparison with the control. In particular, the level of pErk in cells treated with *L. fermentum* MG5091 or MG5159 and the level of pNF-κB in cells treated with *L. fermentum* MG4538, MG5091, MG5159, or *L. paracasei* MG5015 were higher than those in the positive control (LPS). Taken together, our results demonstrate that *Limosilactobacillus* and *Bifidobacterium* affect the immune responses by regulating immune cytokines through the ERK pathway and the transcription factor NF-κB. The *Limosilactobacillus* fractions increase NF-κB activation and TNF-α production in RAW264.7 cells, with protoplasts being most efficient, followed by the cell wall and polysaccharide–peptidoglycan complex [26]. Both crude extract of *L. fermentum* and purified lipoteichoic acid (LTA) significantly induce TNF-α secretion in RAW264.7 cells and spleen cells [27]. Therefore, we speculate that *L. fermentum* MG4538, MG5091, and MG5159 had innate immunity-enhancing effects in RAW 264.7 cells via components such as the protoplast, cell wall, and polysaccharide–peptidoglycan, which were higher than the effects of other strains.

The effects of treatment with *Bifidobacterium* and *Limosilactobacillus* on NK cells and their mechanisms have not been clearly elucidated. NK cells are important for innate immunity; after recognizing target cells, they secrete GrzB and perforin into the intercellular space [28]. GrzB in the cytoplasm of target cells leads to the cleavage and activation of caspases, which induces apoptosis. All seven bacterial strains tested showed increased NK cytotoxicity and secretion of GrzB from NK cells. Treatment with *L. fermentum* MG4538, MG5091, and MG5159 was the most efficient in increasing NK proliferation, NK cytotoxicity, and GrzB expression in comparison with untreated cells (Figure 2A–C). Our results confirmed that the changes in the percentage of the CD56dim and NKG2D populations among NK-92 cells treated with bacterial strains did not match the trends of NK proliferation and NK cytotoxicity. Because not only CD56 and NKG2D but also NKP46 and other receptors affect NK-cell activation [29], we speculate that NK activation may be influenced by other factors.

SCFs secreted by *Lactobacillus* has immune-enhancing effects by affecting the activity of macrophages and NK cells [30]. Therefore, we analyzed the content of representative SCF components acetic acid, propionic acid, butyric acid contained in *L. fermentum*, which showed high immune-enhancing effect in cell experiments. According to our previous study, the acetic acid contents of *L. reuteri* MG505 and *B. lactis* MG741 were 1444.0 and 2610.0 mg/L, respectively [31]. The acetic acid content of *L. fermentrum* MG4538, MG5091, and MG5159 was over 4200 mg/L, higher than other strains. In addition, the butyrate contents in *L. reuteri* MG505, *B. bifidum* MG731, and *B. lactis* MG741 were 0.05, 2.00, and 2.05, respectively. Whereas the three strains of *L. fermentrum* had an average of 3.9 mg/L, much higher than the other strains. In a previous study, *L. fermentum* supplementation in mice beneficially modulated the immune system through stimulation of cytokines and immunoglobulins [32]. Therefore, we selected *L. fermentum* MG4538, MG5091, and MG5159 for in vivo study based on the above in vitro study as well as companies’ capability of the mass production of bacterial strains.

In previous clinical trials, 2 × 10^9^ cells of probiotic *L. fermentum* were orally administered for 12 weeks into Parkinson’s patients [33]. In addition, in several studies, more than 1 × 10^9^ cells of *L. fermentum* were used in animal experiments [34,35]. Therefore, we used the bacterial strains at 1 × 10^9^ CFU/mouse. RG was used as a positive control because it was reported to increase the activity and number of T cells and B cells responsible for acquired immunity by acting on cellular and humoral immunity and modulating cytokine and other activities to enhance specific immune responses [36]. We found no significant differences among the seven different groups in body weight until day 14 or in liver weight (Figure 3A,B), confirming the absence of general toxicity from *L. fermentum*. After intraperitoneal injection of CP on days 15 and 17, all CP injection groups showed dramatic weight loss in comparison with the control group. On day 22, the spleen weight of the CP-only group was significantly lower than that of the control group, and that of the *L. fermentum*-administered group was significantly higher than that of the CP group. WBC, lymphocyte, RBC, and platelet levels were significantly lower in the CP group than in the control group, and *L. fermentum* administration significantly increased the levels of immune cells (Table 3). We confirmed that *L. fermentum* had higher immune-enhancing effects compared to RG. This is consistent with the report by Park & Lee [18] that CP administration reduced WBC, platelet, and HGB levels and that *Weissella cibaria* treatment rescued these levels. Therefore, we confirmed that *L. fermentum* has immune-enhancing effects by affecting the spleen and immune cells.

CP treatment impairs B and NK cell proliferation and decreases hematopoiesis and cytokine production in mice [37]. To determine whether *L. fermentum* strains had other immune-enhancing effects, we isolated splenocytes and analyzed their proliferation, NK cytotoxicity, and the frequencies of CD4+, CD8+ T cells, and B220+ cells. Splenocyte proliferation and NK cytotoxicity against YAC-1 cells were higher in the RG and *L. fermentum* groups than in the CP group, and the 3Mix group showed the highest immune-enhancing effects (Figure 4A,B). The percentages of CD4+ T cells and CD8+ T cells were not significantly different among the groups (Figure 4C,D). On the other hand, the proportion of B220+ cells was higher in the RG and *L. fermentum* groups than in the CP group (Figure 4E). Therefore, *L. fermentum* administration exerts immune-enhancing effects by activating NK and B cells rather than T cells. SCFAs, a major metabolite of probiotics, reportedly alleviate rheumatoid arthritis by regulating the immune response through a positive correlation with the frequency of B cells, not T cells [38]. Since this trend is consistent with our experimental results, we speculate that it may have been influenced by SCFAs.

We also investigated whether *L. fermentum* has other immune-enhancing effects. ConA is an antigen-independent mitogen and can be used as an alternative T cell stimulus [39]. ConA is a quick way to activate transcription factors and cytokine production. Interestingly, we observed that IgM production was decreased and IgG production was increased by ConA treatment (Figure 5A,B). The initial immune response is driven mainly by IgM antibodies, and IgG antibodies generate a secondary immune response and promote opsonization and phagocytosis [40]. We speculate that ConA increased the proliferation and immune response of splenocytes and switched antibody production from IgM to IgG. The production of IgG, TNF-α, and IFN-γ in both ConA-untreated and treated cells were lower in the CP group than in the control group, and administration of *L. fermentum* or RG restored the production (Figure 5C,D). Therefore, *L. fermentum* exerts an immune-enhancing effect through regulation of antibody and cytokine production.

Intestinal tight junctions and enterocytes in the intestinal wall are a major defense mechanism to maintain the entire intestinal tract and immunity [41]. ZO is a cytoskeletal linker protein that interacts with the cytoplasmic peripheral membrane proteins, occludin and claudin, to form strong cross-links and also interacts with the membrane cytoskeleton [42]. A previous study revealed that *L. fermentum* strengthened the intestinal wall by reducing structural damage and inflammatory factor accumulation in mouse intestinal tissue [43,44]. We observed that the intestinal wall was damaged and the expression of occludin and ZO-1 were lower in the CP group than in the control group (Figure 6), consistent with a previous report [45]. However, *L. fermentum* administration did not recover the intestinal barrier damage caused by CP injection or increase the expression of proteins related to tight junctions. Butyrate, a probiotic bacterial metabolite, enhances tight junctions by increasing ZO-1 and occludin protein expression [46,47]. If further research is conducted to find a way to strengthen the intestinal tight junctions and recover intestinal damage, it will be possible to develop more effective immune-enhancing probiotics.

## 5. Conclusions

The current study is the first to report that a mixture of *L. fermentum* strains can restore the host immunity in CP-induced immunosuppressed mice. This study demonstrated that oral administration of *L. fermentum* improves both innate and adaptive immunity by stimulating hematopoietic functions, increasing lymphocyte proliferation, and upregulating the levels of immune-activating cytokines and immunoglobulins in CP-induced immunosuppressed mice. These findings suggest *L. fermentum* as a candidate for functional food substance with immune-enhancing effects.

## Figures and Tables

**Table 1:** The origins and species of the seven bacterial strains.

Origin	Species	Strain
Breast milk Fermented food Food	*Limosilactobacillus reuteri*	MG505
	*Limosilactobacillus fermentum*	MG5159
	*Limosilactobacillus fermentum*	MG5091
Infant feces	*Lacticaseibacillus paracasei*	MG5015
	*Bifidobacterium bifidum*	MG731
	*Bifidobacterium lactis*	MG741
	*Limosilactobacillus fermentum*	MG4538

**Table 2:** Short-chain fatty acid profile of *L. fermentum* MG4538, MG5091, and MG5159.

Strain	Acetic Acid (mg/L)	Propionic Acid (mg/L)	Butyric Acid (mg/L)
*L. fermentum* MG4538	4310.0 ± 31.9	2.07 ± 0.14	3.43 ± 0.00
*L. fermentum* MG5091	4310.0 ± 33.1	2.02 ± 0.15	3.96 ± 0.23
*L. fermentum* MG5159	4239.0 ± 31.5	1.84 ± 0.16	4.10 ± 0.29

**Table 3:** Effects of *L. fermentum* on hematological indices in cyclophosphamide-treated mice.

Hematological Index ^(1)^	Group						
	Control	CP	RG	MG4538	MG5091	MG5159	3Mix
WBC (×10^3^ cells/μL)	1.72 ± 0.27 ^a^	1.00 ± 0.25 ^c^	1.50 ± 0.35 ^abc^	1.09 ± 0.30 ^c^	1.58 ± 0.80 ^abc^	1.12 ± 0.37 ^bc^	1.69 ± 0.56 ^ab^
NEU (%)	24.70 ± 5.97 ^c^	33.37 ± 4.9 ^b^	36.92 ± 7.69 ^ab^	34.25 ± 8.56 ^b^	39.73 ± 9.03 ^ab^	33.95 ± 5.30 ^b^	45.57 ± 8.96 ^a^
LYM (%)	59.90 ± 6.00 ^a^	35.30 ± 10.1 ^b^	40.70 ± 5.88 ^b^	42.23 ± 5.46 ^b^	42.78 ± 5.09 ^b^	42.25 ± 7.06 ^b^	40.52 ± 8.26 ^b^
EOS (%)	6.32 ± 3.28 ^c^	13.43 ± 1.82 ^a^	10.08 ± 2.17 ^b^	10.18 ± 3.19 ^b^	8.80 ± 1.98 ^bc^	9.07 ± 3.34 ^bc^	7.87 ± 2.30 ^bc^
BASO (%)	2.50 ± 1.52 ^b^	7.03 ± 2.81 ^a^	2.82 ± 1.68 ^b^	2.85 ± 0.95 ^b^	2.87 ± 1.61 ^b^	2.55 ± 1.52 ^b^	2.72 ± 0.97 ^b^
RBC (×10^6^ cells/μL)	8.78 ± 0.15 ^a^	7.81 ± 0.53 ^b^	8.05 ± 1.03 ^ab^	8.75 ± 0.62 ^a^	8.67 ± 0.76 ^a^	8.50 ± 0.43 ^ab^	8.31 ± 0.37 ^ab^
HGB (g/dL)	13.52 ± 0.47 ^a^	11.82 ± 0.77 ^b^	12.10 ± 1.51 ^b^	13.28 ± 0.82 ^a^	13.43 ± 1.19 ^a^	12.60 ± 0.63 ^ab^	12.43 ± 0.44 ^ab^
HCT (%)	43.97 ± 2.97 ^a^	36.45 ± 3.81 ^d^	37.93 ± 4.15 ^cd^	42.80 ± 2.27 ^ab^	41.80 ± 3.66 ^abc^	39.37 ± 2.28 ^bcd^	39.88 ± 2.20 ^bcd^
MCV (fL)	48.50 ± 2.48 ^a^	46.27 ± 1.20 ^b^	47.30 ± 1.21 ^ab^	47.20 ± 0.89 ^ab^	47.02 ± 0.49 ^ab^	46.52 ± 0.29 ^b^	48.70 ± 2.28 ^b^
MCH (pg)	15 ± 0.17 ^a^	15.08 ± 0.10 ^a^	15.04 ± 0.14 ^a^	14.90 ± 0.17 ^a^	14.93 ± 0.08 ^a^	14.92 ± 0.12 ^a^	15.07 ± 0.20 ^a^
MCHC (g/dL)	31.77 ± 0.49 ^a^	31.72 ± 0.90 ^a^	31.93 ± 0.88 ^a^	31.80 ± 0.32 ^a^	31.85 ± 0.39 ^a^	31.97 ± 0.25 ^a^	31.83 ± 0.39 ^a^
RDW (%)	17.17 ± 0.54 ^a^	15.65 ± 0.87 ^b^	15.72 ± 1.11 ^b^	16.62 ± 0.57 ^ab^	16.63 ± 0.37 ^ab^	15.65 ± 0.42 ^b^	16.57 ± 2.25 ^ab^
MPV (fL)	7.35 ± 0.21 ^c^	7.80 ± 0.31 ^ab^	8.00 ± 0.25 ^a^	7.67 ± 0.16 ^b^	7.70 ± 0.06 ^b^	7.65 ± 0.29 ^b^	7.77 ± 0.18 ^ab^
PLT (×10^3^ cells/μL)	606.00 ± 224.83 ^a^	224.83 ± 111.4 ^b^	517.00 ± 326.99 ^a^	584.33 ± 326.99 ^a^	530.83 ± 131.94 ^a^	553.67 ± 190.10 ^a^	522.17 ± 118.50 ^a^

^(1)^ WBC: white blood cells, NEU: neutrophils, LYM: lymphocytes, EOS: eosinophils, BASO: basophils, RBC: red blood cells, HGB: hemoglobin, HCT: Hematocrit, MCV: mean corpuscular volume, MCH: mean corpuscular hemoglobin, MCHC: mean corpuscular hemoglobin concentration, RDW: Red blood cell distribution width, MPV: mean platelet volume, PLT: platelet count. All data are means ± SD (*n* = 6) and were analyzed with one-way ANOVA to compare differences between groups for each item. Different letters indicate significant difference between means at *p* < 0.05.

## Data Availability

MDPI Research Data Policies.
